# Structure-Guided Temporin L Analogs Development to Inhibit the Main Protease of SARS-CoV‑2

**DOI:** 10.1021/acsmedchemlett.5c00370

**Published:** 2025-09-16

**Authors:** James Stewart, Ruoqing Jia, Md Ackas Ali, Blaise Williams, Kaylee Stone, Ryan Faddis, Md. Shahadat Hossain, Andrew C. McShan, Mohammed Akhter Hossain, Mohammad A. Halim

**Affiliations:** † Department of Chemistry and Biochemistry, 271594 Kennesaw State University, Kennesaw, Georgia 30144, United States; ‡ School of Chemistry and Biochemistry, 1372 Georgia Institute of Technology, Atlanta, Georgia 30332, United States; § Division of Infectious Diseases and Division of Computer-Aided Drug Design, The Red-Green Research Centre, BICCB, Tejgaon, Dhaka 1215, Bangladesh; ∥ The Florey, University of Melbourne, Melbourne, Victoria 3010, Australia

**Keywords:** Peptide inhibitor, Temporin L, Main Protease, SARS-CoV-2, Molecular Dynamics

## Abstract

Peptide-based inhibitors exhibit considerable potential as antiviral agents targeting SARS-CoV-2. In this study, we designed analogs (TLP-1, TLP-2, and TLP-3) of Temporin L (TL) peptide with the specific objective of selectively interacting with and targeting the main protease (Mpro) of SARS-CoV-2. The synthesis and characterization of TLPs were employed using solid-phase peptide synthesis and LC-MS respectively. CD and solution NMR spectroscopy elucidated the overall structure of the TLPs relative to TL, revealing folded peptides where introduced mutations alter the peptide conformation for binding to Mpro. MD simulations highlighted improvements in TLP’s stability and interactions with Mpro. FRET based protease activity assays provided evidence that TLPs exhibited enhanced inhibitory activity against Mpro. The results of our study reveal the promising prospects of TLPs as attractive candidates for *in vivo* investigations, thereby contributing to the progress of peptide-based therapeutic approaches targeting SARS-CoV-2.

## Graphical abstract

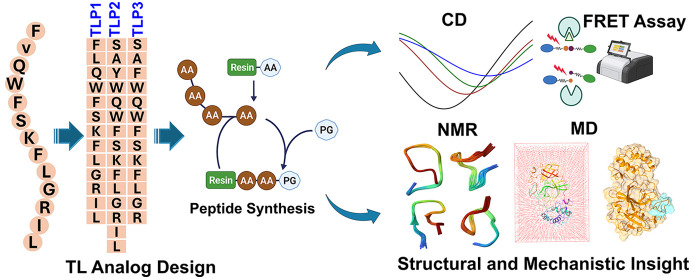

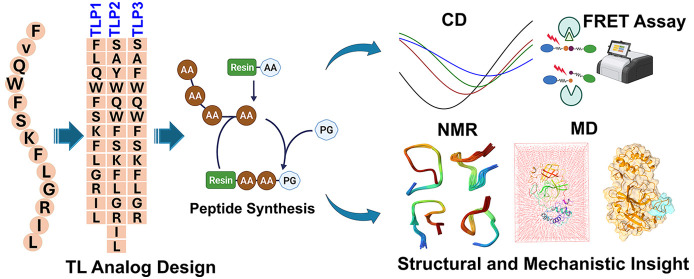

The coronavirus designated severe acute respiratory syndrome coronavirus 2 (SARS-CoV-2) was found to cause respiratory disease of severities ranging from mild or asymptomatic to fatal in infected patients.^1^ SARS-CoV-2 is the cause of the ongoing global pandemic. A member of the genus betacoronavirus of the family coronaviridae, it is an enveloped, positive-sense single-strand RNA virus with a genome size of roughly 30 kbp. Its genome encodes a total of 29 proteins: four structural proteins, nine accessory proteins, and 16 nonstructural proteins (NSPs). The NSPs are initially translated as two polyproteins, PP1a and PP1ab, and are subsequently processed by two viral proteases. The polyproteins PP1a and PP1ab, which contain 16 NSPs, are encoded by ORF1ab. Among these NSPs is NSP5, a chymotrypsin-like protease referred to as 3CL^pro^ (3-chymotrypsin-like protease) or Mpro (main protease), which is responsible for most of the proteolytic processing required to liberate the 16 NSPs contained within the polyproteins.^2^ As these proteins are essential for viral infectivity and genome replication and are not functional until released from the polyprotein, the initial processing of PP1a and PP1ab by 3CL^pro^ represents one of the earliest and most critical steps in viral replication and maturation.

The crystal structure of the 3CL^pro^ enzyme^3,4^ reveals that the protein exists in its catalytically competent form as a homodimer with three domains: two catalytic domains (domains I and II) and one dimerization domain (domain III).^5^ A histidine-cysteine (His41-Cys145) catalytic dyad is present in the active site, and its structure is in general highly conserved among coronaviruses.^6^ 3CL^pro^ performs base-catalyzed hydrolysis of protein substrates wherein the thiol sulfur of Cys145 is deprotonated by His41 and subsequently acts as the nucleophile which attacks the peptide bond of the substrate at the carbonyl group.^7^ Cleavage of target proteins generally occurs at a recognition sequence of Leu-Gln followed by a small amino acid (Ser, Ala, Gly) with peptide cleavage occurring between the Gln and small residue.^4^ The catalytic residues have previously been identified as therapeutic targets for antiviral drug development in studies already conducted against 3CL^pro^ of SARS-CoV-2 as well as other coronaviruses such as MERS-CoV and SARS-CoV.^8,9^ Inhibition of this enzyme is very likely to arrest viral replication, and an antiviral agent that could be designed to bind well to the active site of 3CL^pro^ of SARS-CoV-2 could demonstrate broad-spectrum activity against other coronaviral diseases, as the binding pocket is highly conserved among many coronavirus species.^6^ Importantly, as there is no known human protease with a similar cleavage specificity, such inhibitors are unlikely to be toxic.^4^

Peptide therapeutics present a reasonable avenue for the development of such an antiviral drug.^10–17^ Peptide inhibitors have the potential to possess many advantages as they are highly selective, well tolerated, are generally associated with fewer adverse effects, and undergo faster clinical development and clearance of the FDA approval process.^18^ Existing computational works have investigated the possibility of blocking viral entry into the cell through inhibition of or binding to the receptor-binding domain (RBD) of the spike protein by synthetic peptides.^19,20^ Additionally and more recently, there have also been reports of synthetic peptide inhibitors that target 3CL^pro^.^21,22^ However, the number of reports overall regarding the design of peptide inhibitors that effectively target 3CL^pro^ is relatively small compared to the number of reports that focus on the RBD. Previous study from our group revealed that Temporin L (FVQWFS­KFLGRIL herein referred to as TL), derived from the common frog *Rana temporaria*, can effectively inhibit the main protease of SARS-CoV-2 with moderate inhibition efficacy.^10^

In this study, several Temporin L analogs (TLPs) were designed and optimized. Structures of these analogs were determined by circular dichroism (CD) and solution nuclear magnetic resonance (NMR) spectroscopy, revealing how introduced mutations alter peptide conformation. Molecular dynamics (MD) simulations were employed to examine the binding mode and complex stabilities of TLP with the SARS-CoV-2 Mpro. Fluorescence Resonance Energy Transfer (FRET) based protease activity assays were performed to assess the inhibitory effects of all peptides on Mpro. Together, these results suggest that Temporin L analogs have enhanced Mpro binding and inhibitory capacity relative to wild-type Temporin L and could serve as effective inhibitors against the main protease of SARS-CoV-2.

We previously identified the peptide Temporin L (FVQWF­SKFLGRIL herein referred to as TL), derived from the common frog *Rana temporaria*, as an inhibitor of SARS-CoV-2 Mpro activity.^10^ Temporin L analogs have been previously developed for therapeutic applications, such as antimicrobial and antiviral agents.^23,24^ The peptide analogs had been rationally designed employing the Mpro substrate sequence as a template.^25^ Specific residues in the peptide sequence are replaced with amino acids from the Mpro substrate to improve binding interactions and inhibition. Accordingly, the TLP-1 (FLQWF­SKFLGRIL), TLP-2 (SAYWQW­FSKFLGRIL), and TLP-3 (SAFWQW­FSKFLGR) were synthesized utilizing conventional Fmoc-based techniques for solid-phase peptide synthesis (details are in the Supporting Information). The purity and identity of the synthesized peptides were confirmed by liquid chromatography and mass spectrometry (Figure S1).

To investigate how mutations in TL could alter the fold and/or conformational landscape of TLPs,^26^ we performed circular dichroism (CD) spectroscopy to characterize the peptide secondary structure. TL and TLP-1 were found to be a mix of α-helical and random coil structures with a single dip at ∼203 nm (Figure 1). Interestingly, TLP-2 and TLP-3 exhibited more robust α-helical signals with dips at 208 and 222 nm (Figure 1). These data suggest that mutations introduced into TL result in structural changes in the TLP analogs relative to TL, which could influence their ability to interact with and inhibit Mpro. One limitation is that due to peptide solubility, the organic solvent hexafluoro-2-propanol was included, which has been shown to induce α-helical characteristics into the peptides.^27^

**Figure 1:**
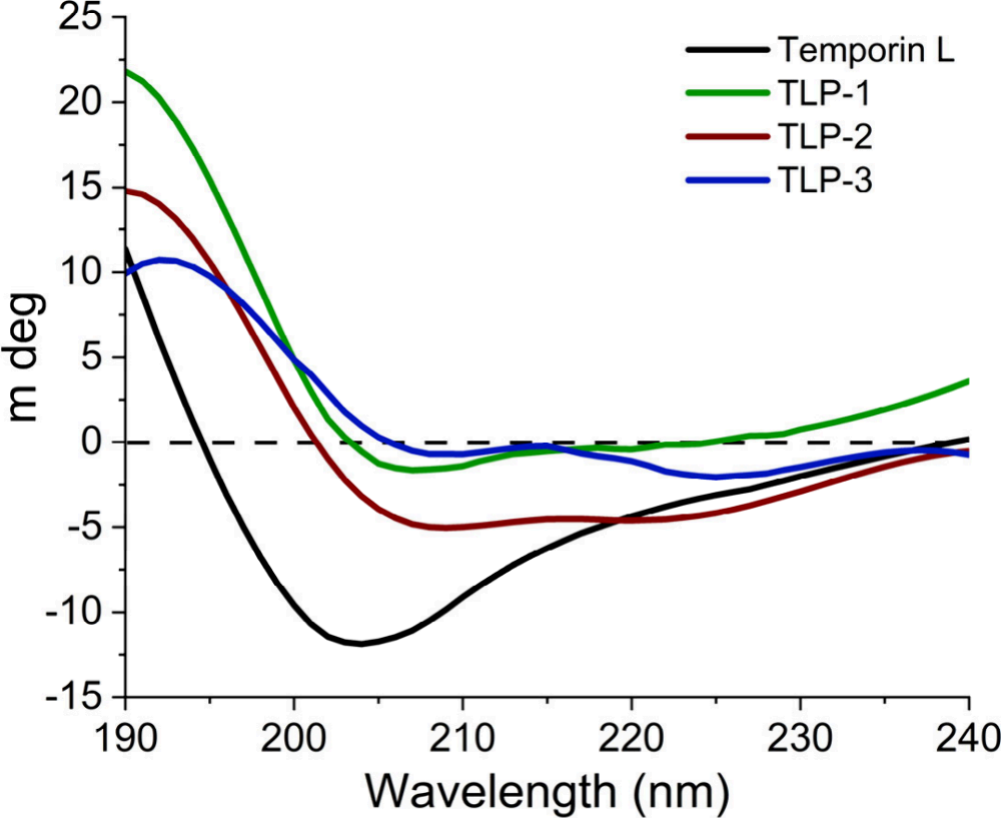
CD spectra of Temporin L, TLP-1, TLP-2, and TLP-3 in phosphate buffer (pH 7.5) with 0.5% hexafluoro-2-propanol (HFP) at 25 °C.

The structure of TL has been previously determined by solution NMR in the presence and absence of detergent.^10,28^ In the presence of detergents, such as sodium dodecyl sulfate (SDS), TL adopts a linear structure with α-helical characteristics. However, in the absence of detergent, TL adopts a folded structure but lacks well-defined α-helical or β-strand characteristics, consistent with CD data. Given CD observations that Temporin L analogs exhibit altered conformations relative to TL, we sought to determine the solution-state structures of TL mutants (TLP-1, 2, 3) to elucidate differences with TL at high-resolution (Figure 2A). Structural determination of Temporin L analogs was achieved using CYANA with the backbone and side-chain chemical shifts (obtained from 2D TOCSY and carbon HSQC experiments) and through space distance restraints (obtained from 2D NOESY experiments). The resulting ensembles of the 10 lowest energy structures of TLP-1, TLP-2, and TLP-3 relative to our previously determined TL structure, all determined in the absence of detergent, are shown in Figure 2B. The heavy-atom RMSD of the ensembles is less than or equal to 1 Å, indicating excellent convergence of the structural calculation for all peptides. All of the NMR structures of the four peptides reveal partially folded peptides lacking α-helical or β-sheet characteristics. The lack of α-helical or β-sheet secondary structural elements is consistent with the TL and TLP-1 CD spectra, which exhibit signals with strongly random coil character. However, the CD spectra of TLP-2 and TLP-3 show α-helical characteristics, while the NMR structures do not contain any secondary structure, which could be due to the use of HFP in CD experiments. We expect that the NMR structures determined here are representative of those found in the solution during the protease inhibition assay described below, since similar buffers were used.

**Figure 2:**
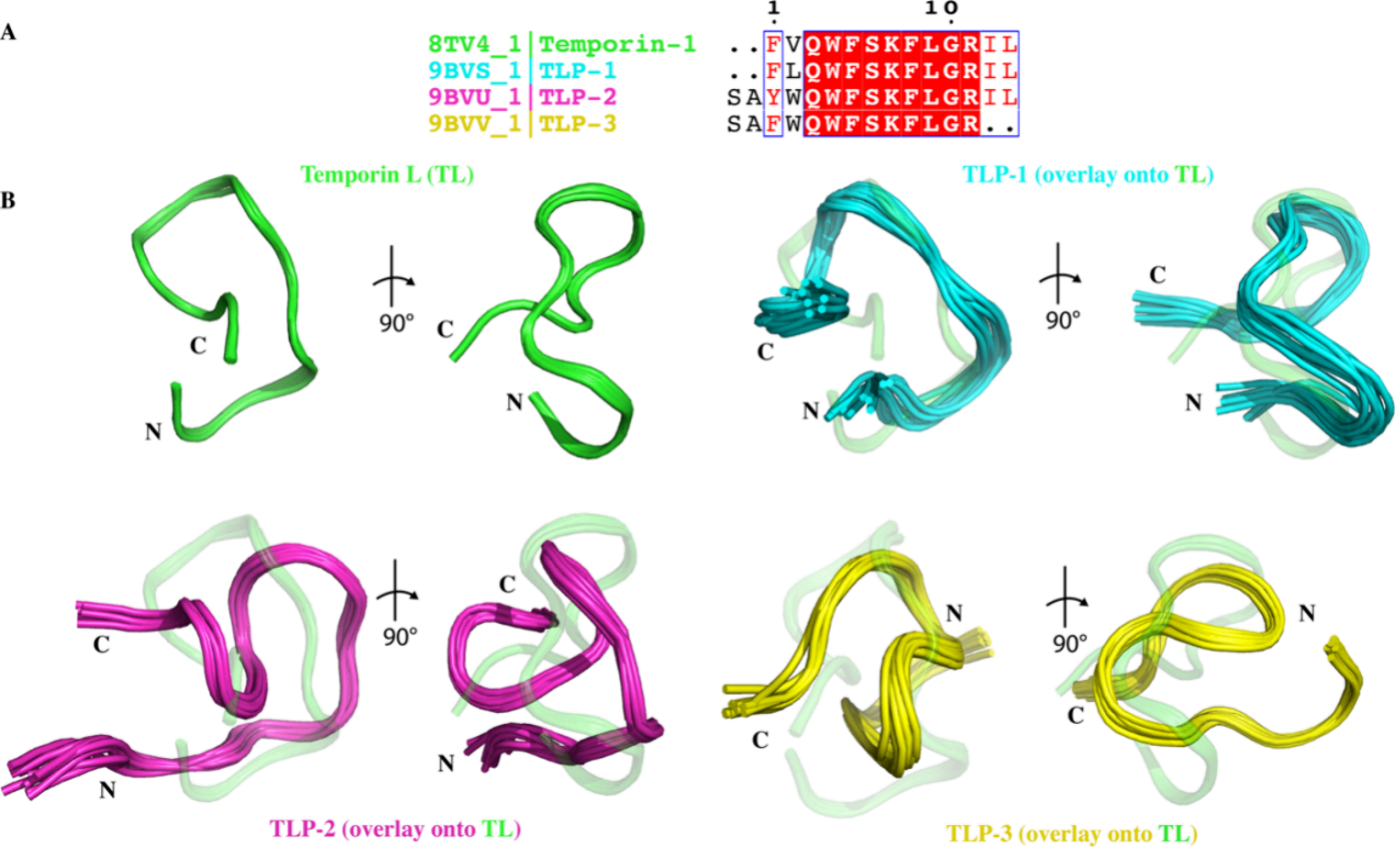
(A) Sequence alignment of TL, TLP-1, TLP-2, and TLP-3 was performed in Clustal Omega and analyzed with ESPript 3. Fully conserved residues are in white text in red boxes; partially conserved residues are shown in red text. (B) Cartoon representation of the front and side view of the 10 lowest energy NMR structures of TL (PDB ID 8TV4),^10^ TLP-1 (PDB ID 9BVS), TLP-2 (PDB ID 9BVU), and TLP-3 (PDB ID 9BVV). For TLP-1, TLP-2, and TLP-3, they are overlaid onto TL (transparent, green). N: N-terminus, C:C-terminus

The N-terminus and C-terminus of TL, TLP-1, and TLP-2 are close in space, which is promoted by a network of polar interactions between residues Lys7/9, Ser6/8, and the hydrophobic tail of TL, TLP-1, and TLP-2 (Ile12 and Leu13). For TLP-3, due to the lack of Ile-Leu at the C-terminus as a result of mutation, the core structure is facilitated by the polar networks between resides GLN5 and Phe7, Gln5 and Ser8, Phe10 and Ser8, Ser1 and Ser8, Ser1 and Lys9, and Ala2 and Lys9. The core of the four peptides is further stabilized by several conserved hydrophobic interactions among residue Ile12, Phe5/7, and Phe8/10 (Figure 3). Comparison of the TL structure with TLP-1 (PDB ID 9BVS), TLP-2 (PDB ID 9BVU), and TLP-3 (PDB ID 9BVV) structures reveals one major change, (Figure 2 and 3). Similar to TL, TLP-1 and TLP-2 have N- and C- terminus close in distance. Apart from that, the N- and C- terminus of TLP-3 are oriented away from each other, presenting a different kind of interaction. We assume the difference is caused by the different amino acid components lying in the sequence of peptides. Based on the sequence alignment results shown in Figures 2A and 3, the Ile-Leu tail was removed from the C-terminus of TLP-3, which is responsible for stabilizing the structure with hydrogen bonding and hydrophobic interactions. Instead of hydrophobic residues, hydrophilic, positively charged Arg at position 11 interacts with Phe7, causing the termini to separate from each other. Together, these results suggest the different Temporin L analogs have difficult structures, which could result in differences in binding to Mpro.

**Figure 3:**
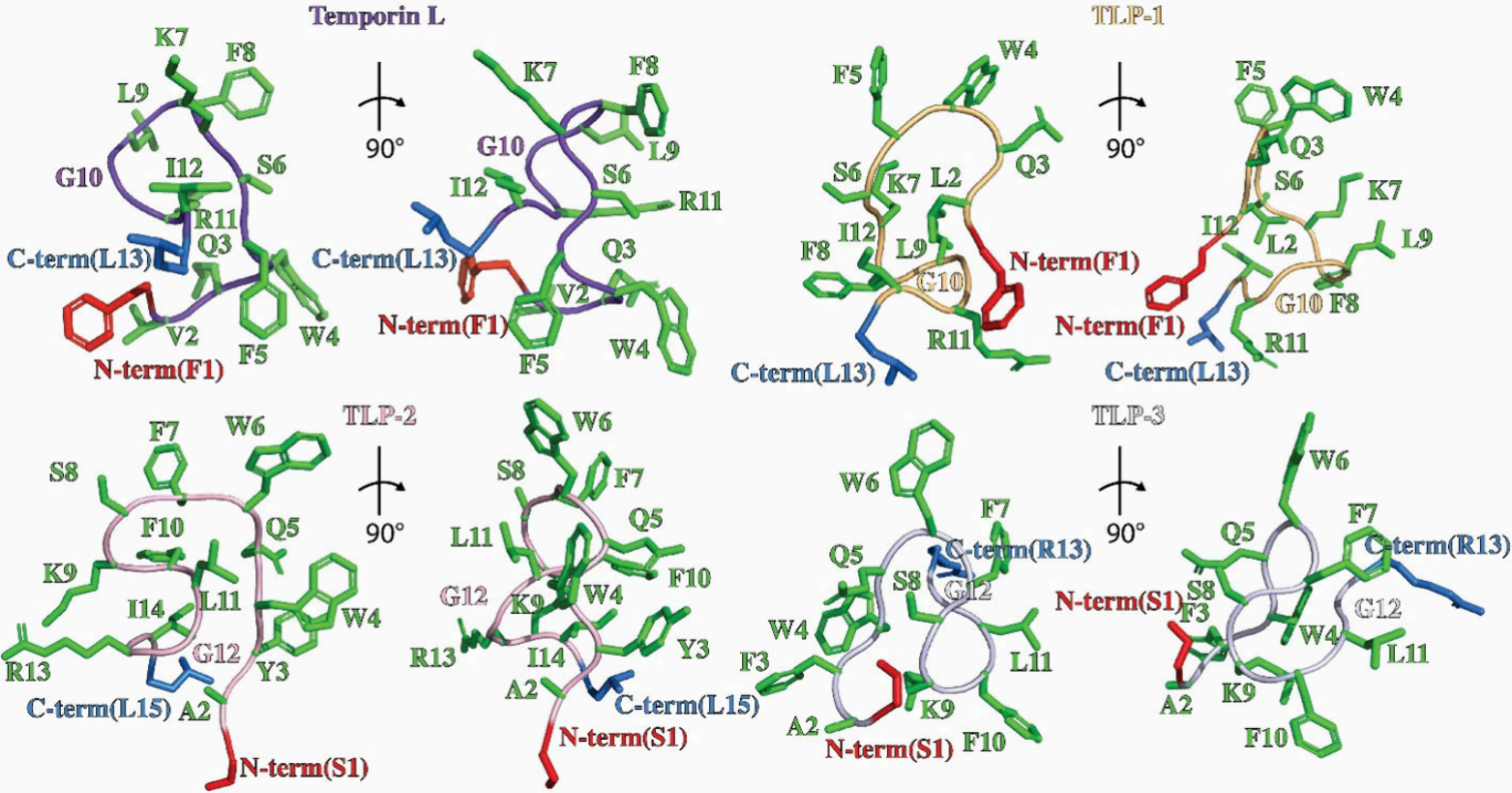
Stick representation of the lowest energy NMR structures of TL, TLP-1, TLP-2, and TLP-3. Hydrophobic residues are colored teal, charged residues are colored purple, and uncharged polar residues are colored pink.

MD simulations (500 ns) of NMR-derived peptide structures with Mpro revealed that all complexes exhibited stability during the simulation period (Movies SM1–SM4). TLP-1 and TLP-3 displayed slightly fluctuating root-mean-square deviation (RMSD) profiles at the beginning. After 300 ns, these two complexes showed a decreasing RMSD trend, with TLP-3 mostly aligning with the apo and TLP complexes and TLP-1 aligning with the TLP-2 peptide (Figure 4A). The radius of gyration (Rg) profiles of all peptides were similar and stable over the simulation period, except for TLP-3. The Rg fluctuation in TLP-3 might be attributed to the C-terminal instability of the peptide, as observed in the TLP-3 trajectory movie analysis. After 400 ns, all peptide complexes merged, indicating that they reached a stable profile and similar occupancy of the binding pocket (Figure 4B). Evaluation of the solvent-accessible surface area (SASA) revealed a substantially decreased profile for TLP-3 compared to the other complexes (Figure 4C). Furthermore, analysis of individual protein residue fluctuations during the MD simulations revealed that TLP-3 induced lower root-mean-square fluctuation (RMSF) values in critical regions compared to the other peptides, while the highest fluctuation occurred in the apo protein (Figure 4D).

**Figure 4:**
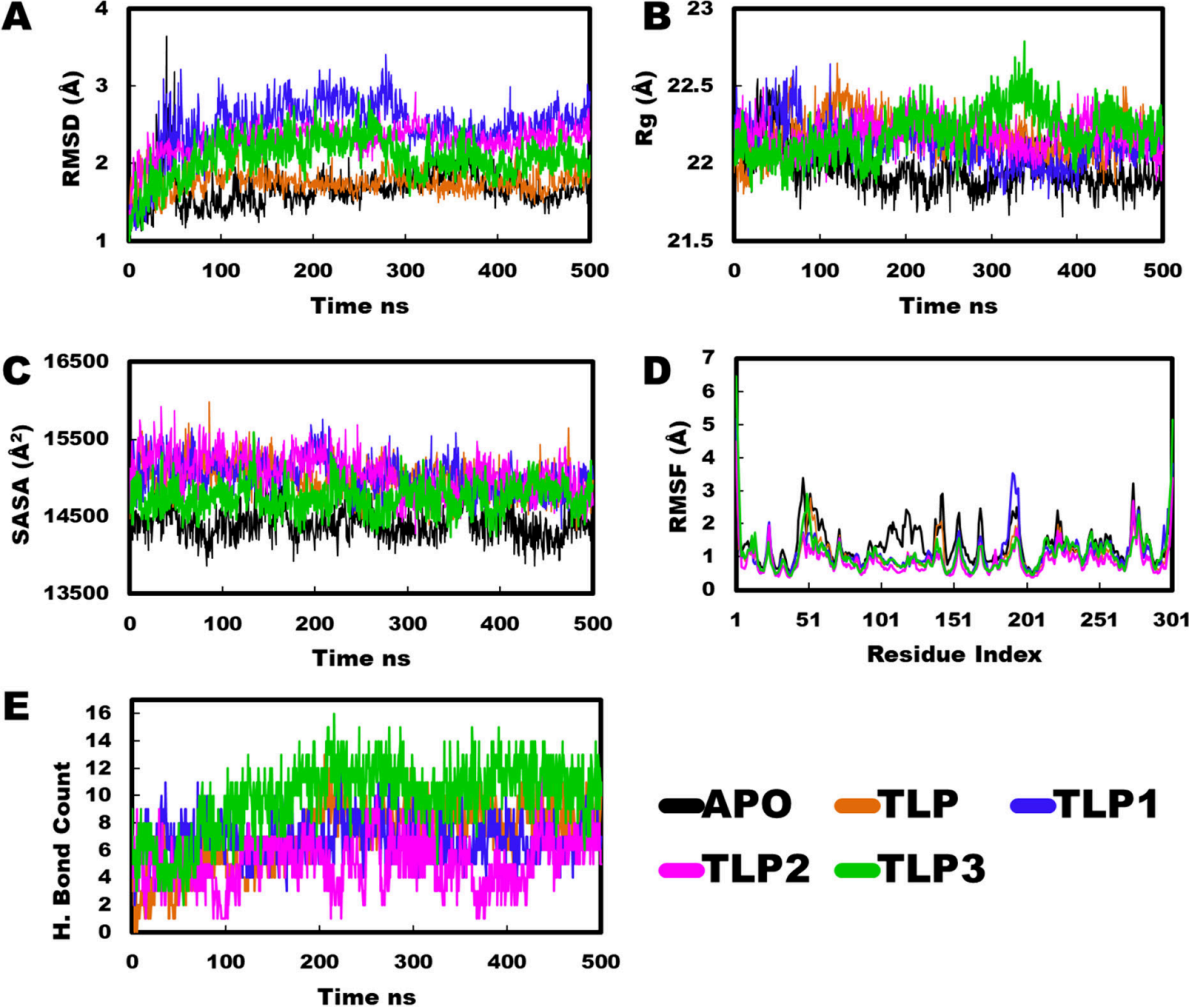
(A) Root Mean Square Deviation (RMSD); (B) Radius of Gyration (Rg); (C) Solvent Accessible Surface Area (SASA); (D) Root Mean Square Fluctuation (RMSF); and (E) Hydrogen Bond Counts.

To check the protein stability, we determined interdomain distance. Peptide binding resulted in a reduction of the interdomain distance between Domains I and II relative to the apo protein, a conformational adjustment that is essential for maintaining the optimal spatial arrangement of the catalytic residues His41 (Domain I) and Cys145 (Domain II), thereby facilitating enzymatic activity. Minor variability was observed between Domains II and III, while the spacing between Domains I and III remained largely unchanged (Figure S7). Clustering analysis of the simulation trajectories demonstrated that, for TL, TLP, TLP-1, and TLP-3, the most populated cluster accounted for over 40% of frames and the top three clusters together covered >90% of the trajectory, indicating structural persistence of the bound state. In contrast, TLP-2 exhibited a broader distribution across clusters, reflecting greater conformational variability (Figure S8), which is also supported by the trajectory movies. These analyses demonstrate that peptide binding stabilizes Mpro by preserving a catalytically favorable Domain I–Domain II arrangement, except in TLP-2, which shows greater conformational variability.

The evaluation of hydrogen bond counts provides crucial insights into the specificity and strength of interactions within the protein-peptide complex. TLP-3 consistently exhibited a higher hydrogen bond count compared with other complexes over the entire simulation period. Although all complexes, except TLP, were entirely buried within the binding pocket, TLP-3 interestingly exhibited the highest number of hydrogen bond interactions (Figure 4E).

Several binding pocket residues of Mpro, including Glu166, Gln189, Asp48, Ser144, Gly143, Thr24, and Cys44, played pivotal roles in peptide binding and stabilization for TLP-3. These residues demonstrated the highest interaction fractions of 2.5, 2.5, 2, 1.4, 1, 0.9, and 0.8, respectively, as shown in Figure 5A and C. Analysis of the average distance (∼3Å) revealed significant hydrogen bonding between these residues and TLP-3, with Glu166, Gln189, and Asp48 exhibiting multiple contacts with peptide residues (Figure S2). Among the interacting residues, Glu166, Gln189, Thr24, Cys44, and Asp48 interacted with the peptide for more than 50% of the simulation period (Figure S2).

**Figure 5:**
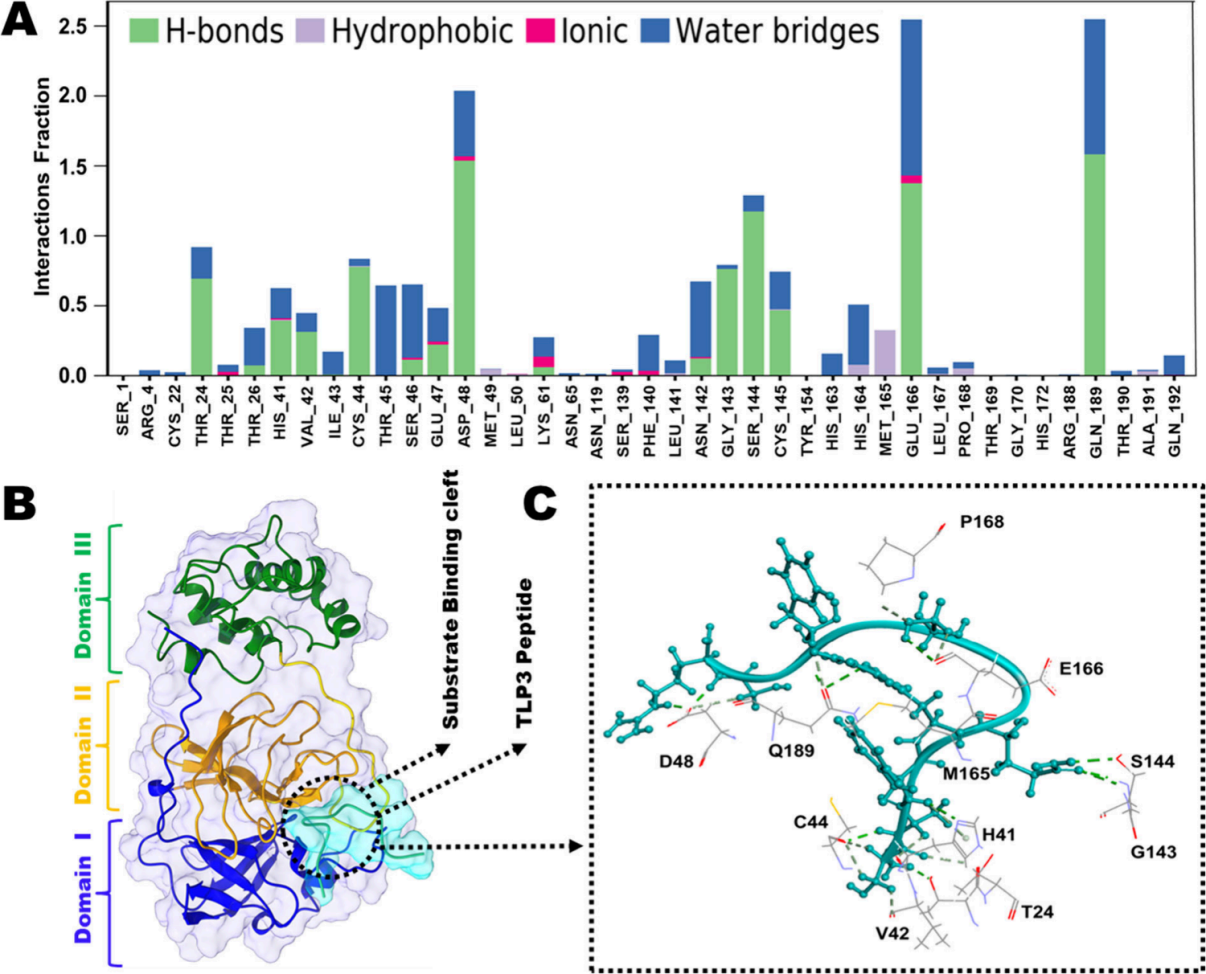
(A) Contributing percentage interactions of TLP-3 with the Mpro residue (C terminal fewer interactive residues were omitted). (B) Binding mode of TLP-3 peptide with Mpro. (C) Residues of Mpro associated with peptide binding (H bonds only).

Furthermore, a subset of residues, namely, Met165, His164, and Met49, displayed hydrophobic interactions with the peptide, along with some ionic interactions involving Lys61, Glu166, and Phe140 residues (Figure 5A). TLP-3 established a strong hydrogen bond with the catalytic residue HIS41 at a distance of 3Å, while another catalytic residue engaged in hydrophobic interactions at a distance of 4.5Å. TLP exhibited more interaction profiles compared to TLP-1 and TLP-2, despite TLP not being entirely buried within the binding pocket (Movies SM1–SM4 and Figures S3B, S4B, S5B). Residues of Mpro, namely Gln189, Glu166, Asp262, and Gln192, showed more than 1.5 interaction fractions with TLP (Figure S3A and C). Additionally, TLP-2 showed interactions with Glu166, Asn142, His164, Glu49, and the catalytic residue His41, with interaction fractions exceeding 1 (Figure S5A and C). TLP-1 exhibited a high interaction fraction with a single residue, Asn142, which was 4. Other residues, such as Glu166, Phe140, and catalytic residue His41, exhibited interaction fractions greater than 1­(Figure S4A and C).

The inhibition efficiency of analogs on the Main protease (Mpro) was tested using a FRET-based protease activity assay. The findings indicated that all analogs exhibited substantial inhibitory efficacy against the Main protease. Among the peptides investigated, TLP-3 demonstrated the most inhibitory effect, as evidenced by its IC_50_ value of 7.0 ± 1.5 μM. When comparing the compounds Temporin L, TLP-1, and TLP-2, their respective IC_50_ values were found to be 39.0 ± 4.5 μM, 11.4 ± 1.5 μM, and 11.5 ± 1.0 μM shown in Figure 6. The involvement of distinct amino acids in TLP-3, specifically Phe and Trp, may contribute to its heightened inhibitory efficacy. We also investigated the cellular viability of TL and TLPs. The CC50 values for Temporin L peptides were determined to be 6.78 μM. The CC_50_ values presented in Figure S6 for TLP-1, TLP-2, and TLP-3 were determined to be 4.65 μM, 2.56 μM, and 14.91 μM, respectively. TLP-3 demonstrated a slightly higher CC_50_ value in comparison to those of TLP-1 and TLP-2.

**Figure 6:**
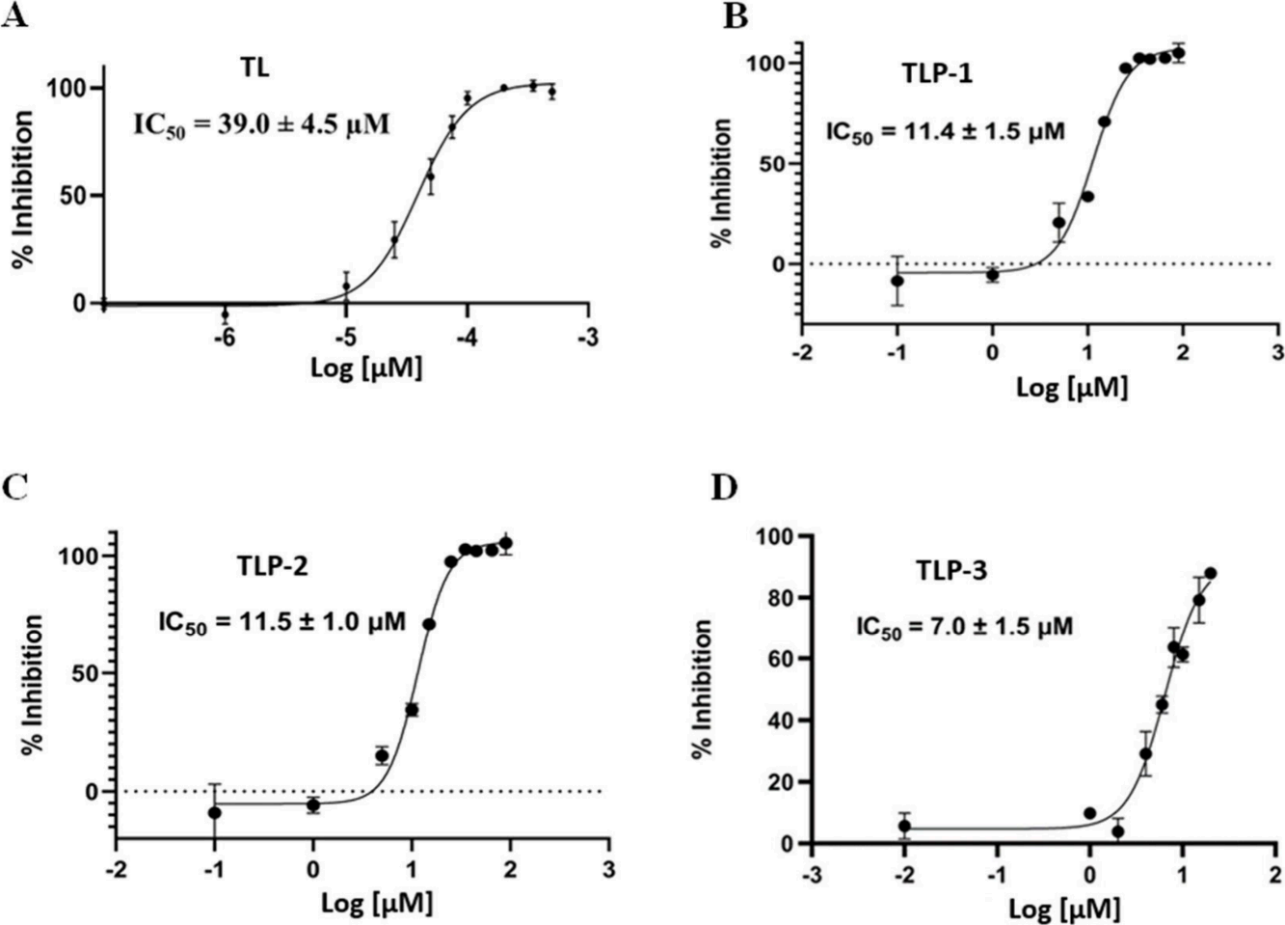
Dose–response curves from a FRET-based protease activity assay of Mpro inhibition with TL and its analogs. (A) TL, (B) TLP-1, (C) TLP-2, and (D) TLP-3 peptides. Error bars are mean ± standard deviation for triplicate experiments (*n* = 3).

The results of our experimental and theoretical study highlight the potential of TLP analogs as viable candidates for further investigation and advancement as a potent inhibitor against SARS-CoV-2. The study utilizes a rational design strategy that can be used as a framework for optimizing peptide therapeutics and improving their safety and efficacy profiles. The peptide exhibits advantageous binding interactions, stability, strong inhibitory properties, and reduced cytotoxicity, rendering it a promising contender for the advancement of peptide-based therapeutics targeting SARS-CoV-2. In the future, the potential for enhancing and perfecting TLP with staple analogs holds promise in facilitating the development of peptide-based therapeutic interventions that are both safer and more efficacious in combating COVID-19 and other diseases mediated by similar viral proteases.

## Supplementary Material











## Notes

Methods related to Fmoc peptide synthesis, LC-MS, CD, solution NMR, molecular dynamics simulation, FRET and cytotoxicity Assay, LC-MS spectra of the synthesized peptides, peptide-protease contact and interactions from MD simulations, cell viability of peptides (PDF)
MD stimulation of Temporin L (MP4)
MD stimulation of TLP-1 (MP4)
MD stimulation of TLP-2 (MP4)
MD stimulation of TLP-3 (MP4)

## Notes

Atomic coordinates and chemical shift assignments for Temporin L analogs have been deposited into the Protein Data Bank (PDB) and Biological Magnetic Resonance Bank (BMRB), respectively with the following accession IDs: TLP-3 (PDB ID 9BVV, BMRB ID 31175), TLP-2 (PDB ID 9BVU, BMRB ID 31174), and TLP-1 (PDB ID 9BVS, BMRB ID 31173). TL was previously deposited with PDB ID 8TV4 and BMRB ID 31100.

## Notes

Safety: No unexpected or unusually high safety hazards were encountered.

## Abbreviations

3CLpro: 3-Chymotrypsin-like Protease
CD: Circular Dichroism
CYANA: Combined Assignment and Dynamics Algorithm for NMR Applications
FRET: Fluorescence Resonance Energy Transfer
HSQC: Heteronuclear Single Quantum Coherence
LCMS: Liquid Chromatography–Mass Spectrometry
Mpro: Main Protease
NMR: Nuclear Magnetic Resonance
NSP: Nonstructural Protein
NOESY: Nuclear Overhauser Effect Spectroscopy
PP: Polyprotein
RBD: Receptor-Binding Domain
RMSD: Root Mean Square Deviation
RMSF: Root Mean Square Fluctuation
Rg: Radius of Gyration
SARS-CoV-2: Severe Acute Respiratory Syndrome Coronavirus 2
SDS: Sodium Dodecyl-Sulfate
SASA: Solvent-Accessible Surface Area
TL: Temporin L
TOCSY: Total Correlation Spectroscopy
