# Supplementary material for: Structure-Guided Temporin L Analogs Development to Inhibit the Main Protease of SARS-CoV‑2
Source: ACS Med Chem Lett. 2025 Sep 16;16(10):1963–70. doi: 10.1021/acsmedchemlett.5c00370 (PMC12516397; doi:10.1021/acsmedchemlett.5c00370)
Supplement: Supplementary file 1 [file ml5c00370_si_001.pdf]

Structure-guided Temporin L Analogs Development to Inhibit the Main Protease of SARS-CoV-2

James Stewart[1‡], Ruoqing Jia[2‡], Md Ackas Ali[1], Blaise Williams[1], Kaylee Stone[1], Ryan Faddis[1], Md. Shahadat Hossain[3], Andrew C. McShan[2], Mohammed Akhter Hossain[4], Mohammad A. Halim[1]*

[1]*Department of Chemistry and Biochemistry, Kennesaw State University, Kennesaw, GA, 30144 USA*

[2]*School of Chemistry and Biochemistry, Georgia Institute of Technology, Atlanta, GA, 30332, USA*

[3]*Division of Infectious Diseases and Division of Computer-Aided Drug Design, The Red-Green Research Centre, BICCB, Tejgaon, Dhaka, Bangladesh*

[4]*The Florey, University of Melbourne, Melbourne, Victoria 3010, Australia*

‡These authors contributed equally to this work.

Corresponding Author: mhalim1@kennesaw.edu

Fmoc Peptide Synthesis, LC-MS, and CD spectroscopy

Peptides (TLP-1, TLP-2, TLP-3) were synthesized on a Rink resin with Fmoc-protected amino acids (CEM, Charlotte, NC, USA). The synthesis was carried out using a Liberty Blue Peptide Synthesizer (CEM), leading to the synthesis of peptides. Following synthesis, the peptides were deprotected and detached from the resin using a high TFA cleavage cocktail (95% TFA, 2.5% H2O, 2.5% triisopropylsilane). The resin was dispersed into the cocktail solution and stirred at room temperature for three hours. After filtration and removal of resin, the peptides were precipitated into cold diethyl ether. The peptides were dissolved in 10%-50% acetic acid and lyophilized under a high vacuum. The crude peptides were purified by reversed-phase column chromatography on an Agilent 1290 Infinity II HPLC system with a 12-minute short-gradient of acetonitrile/water (both 0.1% FA) Purity of each peptide was verified from LTQ XL mass spectrometry (MS). The LC and MS parameters details have been described in our previous study.[10] The secondary structure of the peptides was assessed using circular dichroism (CD) experiments performed on a J-1500 spectropolarimeter. Peptide samples for these experiments were prepared at 50–200μM concentrations in 1,1,1,3,3,3-hexafluoro-2-propanol (HFP), hence the final concentration of HFP was less than ~0.5%.

Solution NMR spectroscopy

The solution NMR structure of Temporin L (TL) was determined previously.[10] TLP-1 (FLQWFSKFLGRIL), TLP-2 (SAYWQWFSKFLGRIL), and TLP-3 (SAFWQWFSKFLGR) peptides (at >95%) purity were dissolved to 50 mM NaCl, 20 mM sodium phosphate, pH 5, 10% D_2O, 5% DMSO-d_6). The addition of 5% DMSO-d_6 was required for high-resolution NMR experiments due to limited peptide solubility. NMR spectra were acquired on a Bruker AVIII HD

800-MHz spectrometer (Bruker, Coventry, UK) equipped with a triple-resonance inverse (TCI) cryoprobe. Experiments were recorded at a temperature of 277.15K. Standard Bruker 2D ^{1}H–^{1}H total correlation spectroscopy (TOCSY) (pulse sequence dipsi2esgpph), 2D ^{1}H–^{1}H nuclear Overhauser enhancement spectroscopy (NOESY) (pulse sequence noesyesgpph), and 2D ^{1}H–^{13}C carbon heteronuclear single quantum coherence (HSQC) (pulse sequence hsqcetgpsi) were acquired with water suppression using an excitation sculping sequence with gradients. Parameters were listed below: ^{1}H 90° pulse length of 7.56 µs, TOCSY mixing time was 80 ms, NOESY mixing time was 300 ms, and relaxation delay was 1.2 s. A total of 2048 data points were recorded in the direct dimension and 512 data points in the indirect dimension. Sparky (version 3.190) was used to perform chemical shift assignments[23]. CYANA software (version 2.1) was used for structural calculation.[24] Intra-proton nuclear Overhauser effects (NOEs), automatically assigned by CYANA software and confirmed manually, were used as distance restraints in the structural calculation. Through simulated annealing protocol, CYANA software generated a total of 200 structures, and ten structures with the lowest energy were selected to produce the final NMR ensemble. In the case of ambiguous NOE assignments by CYANA software, the annealing protocol was applied to determine how close the peaks are based on their volume and heights (determined by Sparky). Images of structures were generated with PyMOL (version 2.5.5). The intramolecular networks were determined by Arpeggio (https://biosig.lab.uq.edu.au/arpeggioweb).[25]

Molecular Dynamic Simulation

The NMR-derived peptide 3D structures were used for further binding mode exploration through molecular dynamics (MD) simulation. ZDOCK server[26,27] was used to molecularly dock the NMR retrieved peptides to Mpro, focusing on the active site residues, based on the RCSB Protein Data Bank (PDB) crystal structure of SARS-CoV-2 Mpro (PDB ID: 6Y2G).[28] Molecular dynamics simulations were performed using the Desmond module of the Schrödinger software package (Schrödinger Release 2020-1: Desmond molecular dynamics system, D. E. Shaw Research, New York, NY, 2020), executed within Maestro. Protein-peptide dock complexes were positioned within an orthorhombic water box, with a buffer distance of 10 Å, and solvated using TIP3P water models. Additional Na+/Cl- ions were introduced to neutralize the systems, and a salt concentration of 0.15 M NaCl was added to replicate the human physiological conditions. Short-range van der Waals and Coulomb interactions were considered within a cutoff radius of 9.0 Å. For the calculation of long-range electrostatic interactions, the particle-mesh Ewald method was employed. Each solvated system underwent energy minimization and equilibration using the default protocol of Desmond in Maestro. An additional 5 ns of minimization was conducted to enhance structural stability before proceeding with the final MD simulation. A 500 ns simulation was performed for each equilibrated system under periodic boundary conditions in the NPT ensemble, employing the OLPS3e[29] force field. The temperature was maintained at 300 K using the Nosè–Hoover chain thermostat, while pressure control at 1 atm was achieved using the Martyna–Tobias–Klein barostat method[30]. A time step of 2 fs was employed, and the coordinates were saved in the trajectory file at intervals of 500 ps. Finally, simulation data was extracted from the saved trajectory using the simulation interaction diagram and simulation event analysis tools available in Maestro.

Principal Components Analysis (PCA) was employed to reveal hidden structural and energy profiles within different groups. Here considered factors such as bond distances, angles, Coulomb, Torsion, Van der Waals energies, and total energy. The PCA analysis utilized MD trajectory data from the last 100 ns of Mpro-Apo and peptide-protein complexes, reducing the multivariate factors into two new matrices by this equation given below-

$$X = T_k P_k^T + E$$

Here, T_k (scores matrix) and P_k^T, (loadings matrix), with k representing the number of factors in the model. The unmodeled variance was captured by the E matrix.

FRET and Cytotoxicity Assay

A fluorometric assay was carried out to assess the effect of the peptides on the activity of the SARS-CoV-2 main protease (Mpro). A peptide substrate (Dabcyl-Lys-SARS-CoV2 Replicase pp1ab (3235-3246)-Glu-EDANS, CPC scientific) was used of enzyme activity upon cleavage of this FRET substrate by the Mpro. Peptides were dissolved in dimethyl sulfoxide (DMSO), maintaining the final DMSO concentration at 0.5%. For an assessment of the TLP peptide's effects on the activity of SARS-CoV-2's Mpro, 11 distinct concentrations (ranging from 200 to 0.01625 µM) were used. The obtained fluorescence readings reported as relative fluorescence units (RFU), were converted to percentage activity values. Finally, GraphPad Prism® software was used to perform a dose-response analysis, enabling the determination of the IC_{50} values for each peptide.

Vero E6 cells from the American Tissue Culture Collection (ATCC) were utilized to carry out the cell viability test.[31] The cells were grown in Dulbecco's modified Eagle's medium (DMEM), which contained phenol red as an added component. Both the temperature and the environment were kept at 37 °C and 5% CO_2, respectively. Trypsin-EDTA diluted to 0.25% was used for the collection of cells. Cells were stained with propidium iodide, and their viability was determined with the help of a Coulter Epics Flow Cytometry Analyzer (Beckman, California, United States). The stock solutions of the peptides were made by dissolving them at a concentration of 1 mM in DMSO. The final amounts of DMSO were kept at around 0.5% throughout the entire process. A 96-well plate was filled with Vero E6 cells with an average density of 10,000 cells per well in 50 µL of assay media. The dish was then incubated. After an initial period of incubation lasting 24 hours, the cells were then treated with peptides at several concentrations that ranged from 33 M to 0.5 M. This was followed by an additional period of incubation lasting 72 hours. The luminescence measurements were taken with a plate reader made by PerkinElmer (PerkinElmer, Massachusetts, United States).

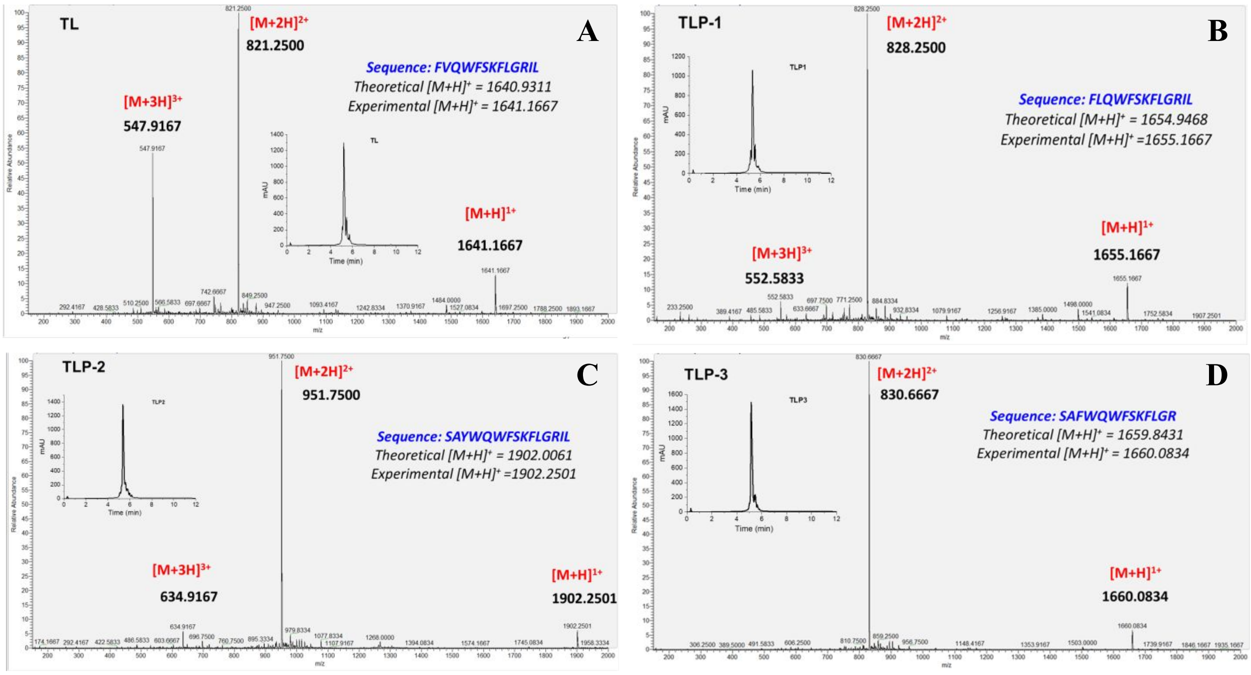

Figure S1: LC-MS validation of Fmoc synthesized peptides. **(A)** TL **(B)** TLP-1 **(C)** TLP-2 and **(D)** TLP-3

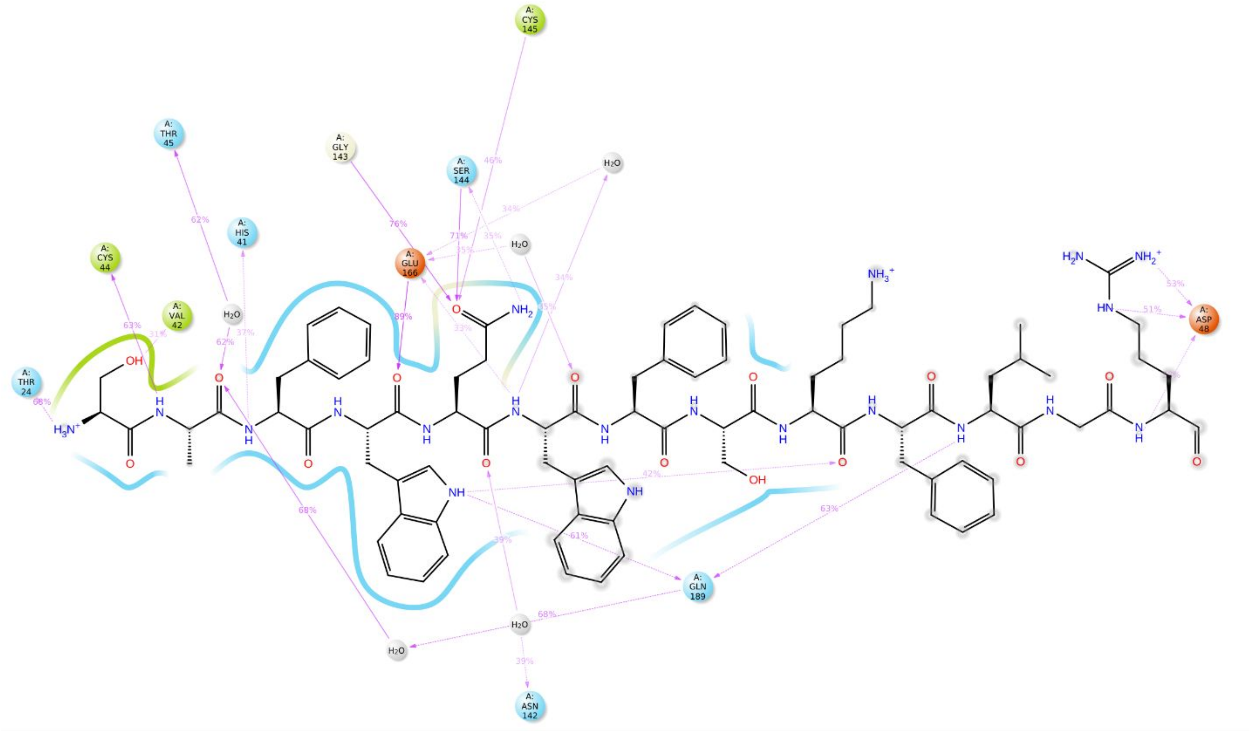

Figure S2: Protein-peptide contact of TLP-3 over the simulation period (only residues that are in contact more than 30% of the time are visualized)

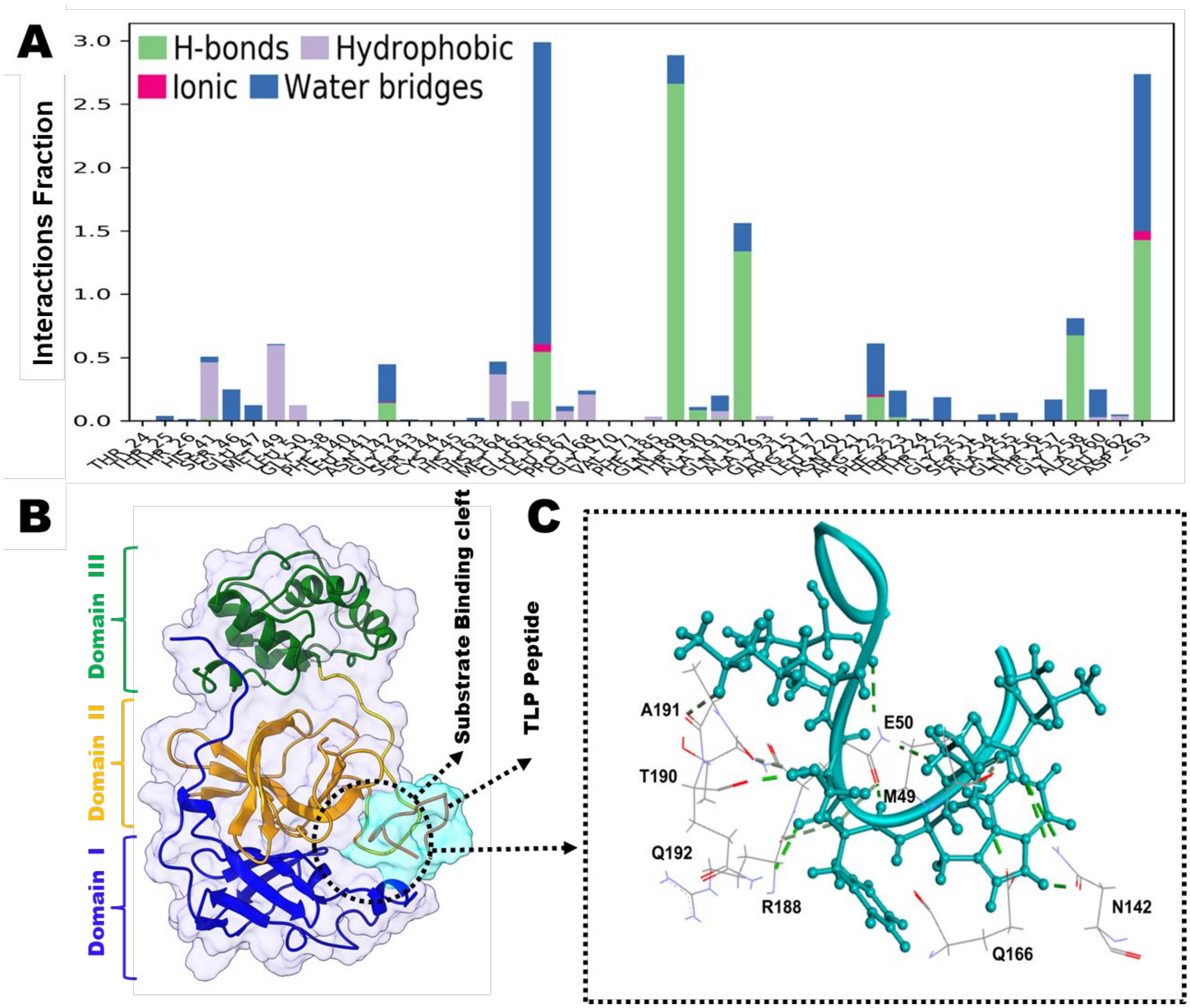

Figure S3: (A) Contributing percentage interactions of TLP with the Mpro residue **(B)** Binding mode of TLP peptide with Mpro **(C)** Residues of Mpro associated with peptide binding (H. bonds only)

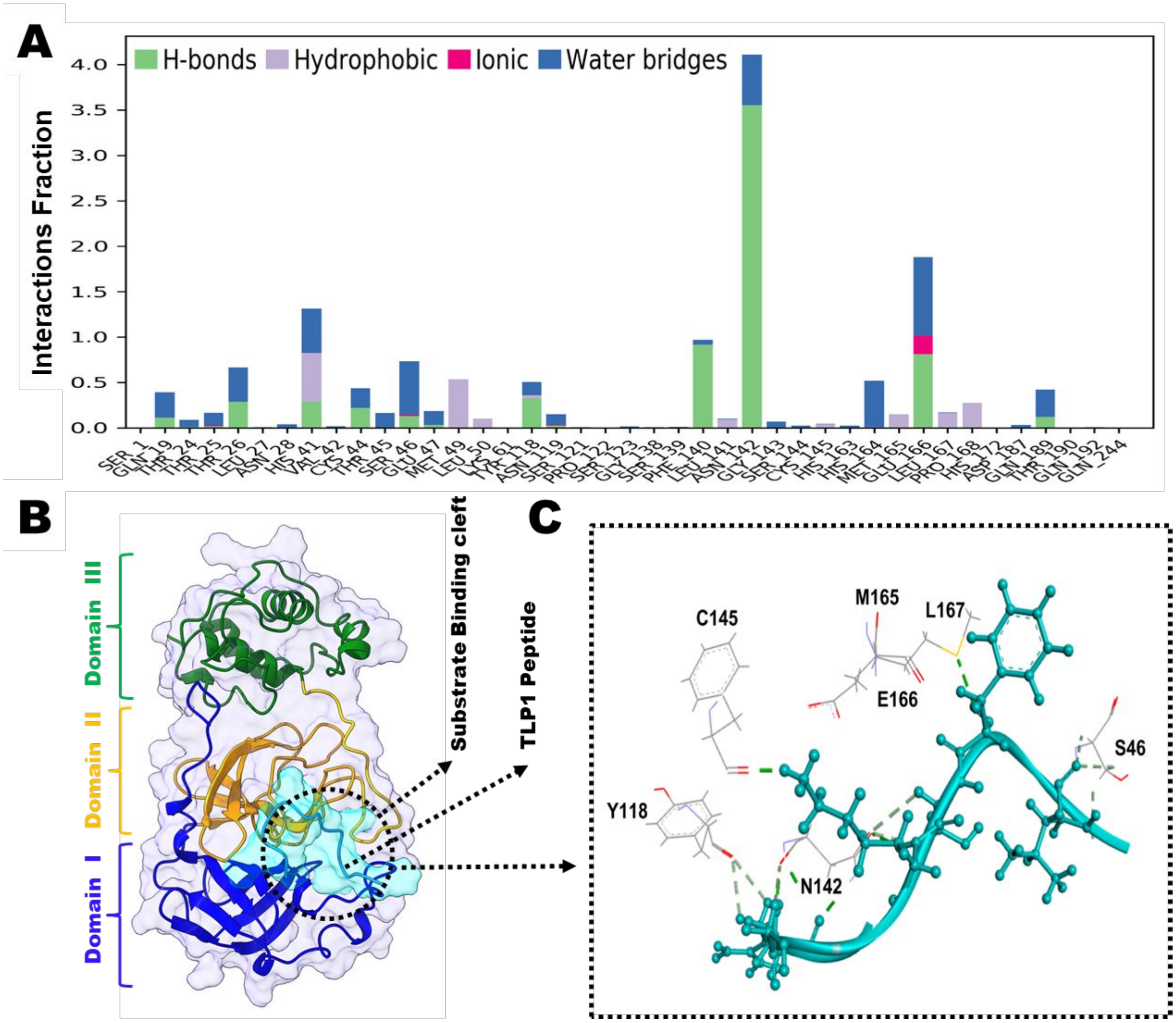

Figure S4: (A) Contributing percentage interactions of TLP-1 with the Mpro residue **(B)** Binding mode of TLP-1 peptide with Mpro **(C)** Residues of Mpro associated with peptide binding (H. bonds only)

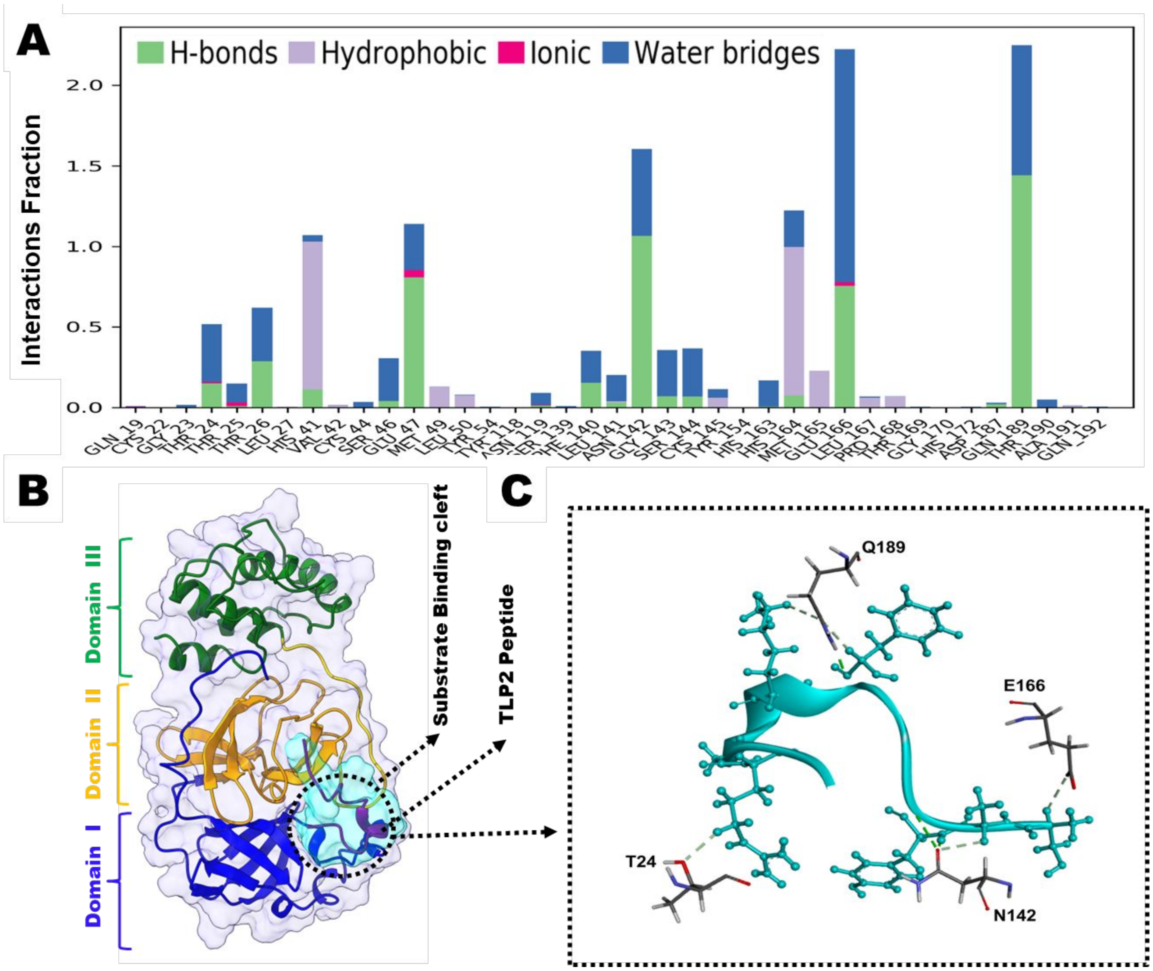

Figure S5: (A) Contributing percentage interactions of TLP-2 with the Mpro residue **(B)** Binding mode of TLP-2 peptide with Mpro **(C)** Residues of Mpro associated with peptide binding (H. bonds only)

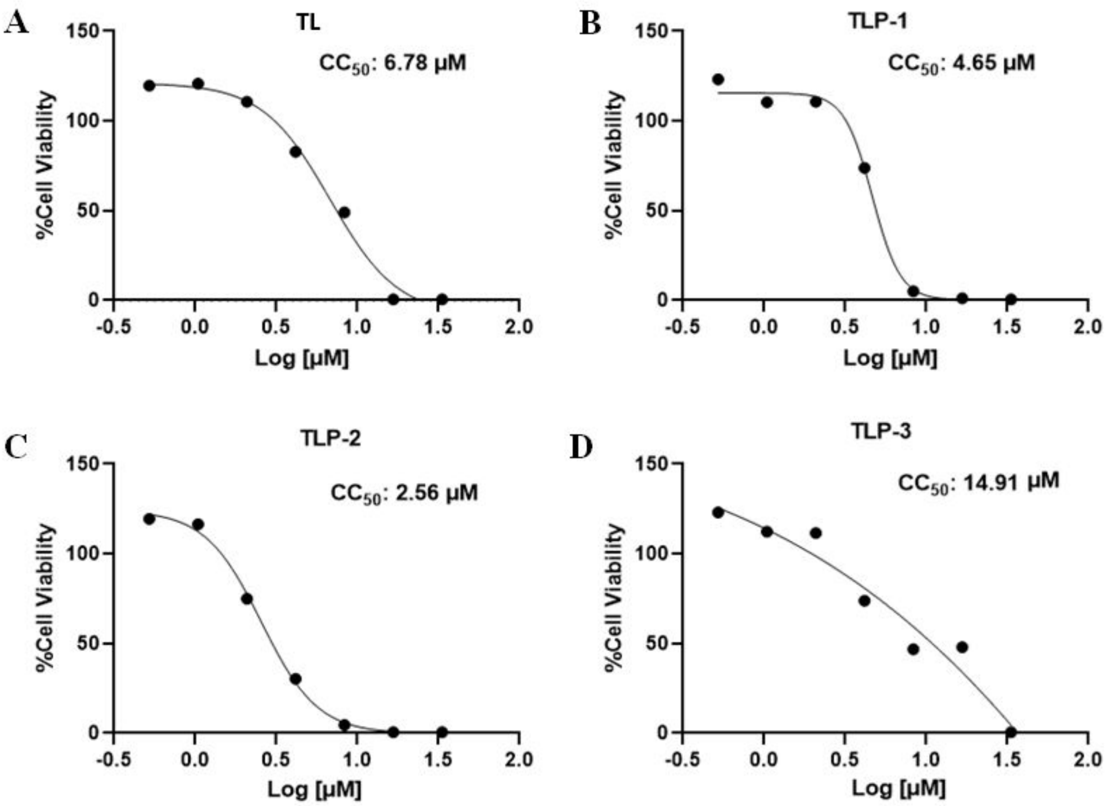

Figure S6. The cell viability of **(A)** TL, **(B)** TLP-1, **(C)** TLP-2, and **(D)** TLP-3 peptides.

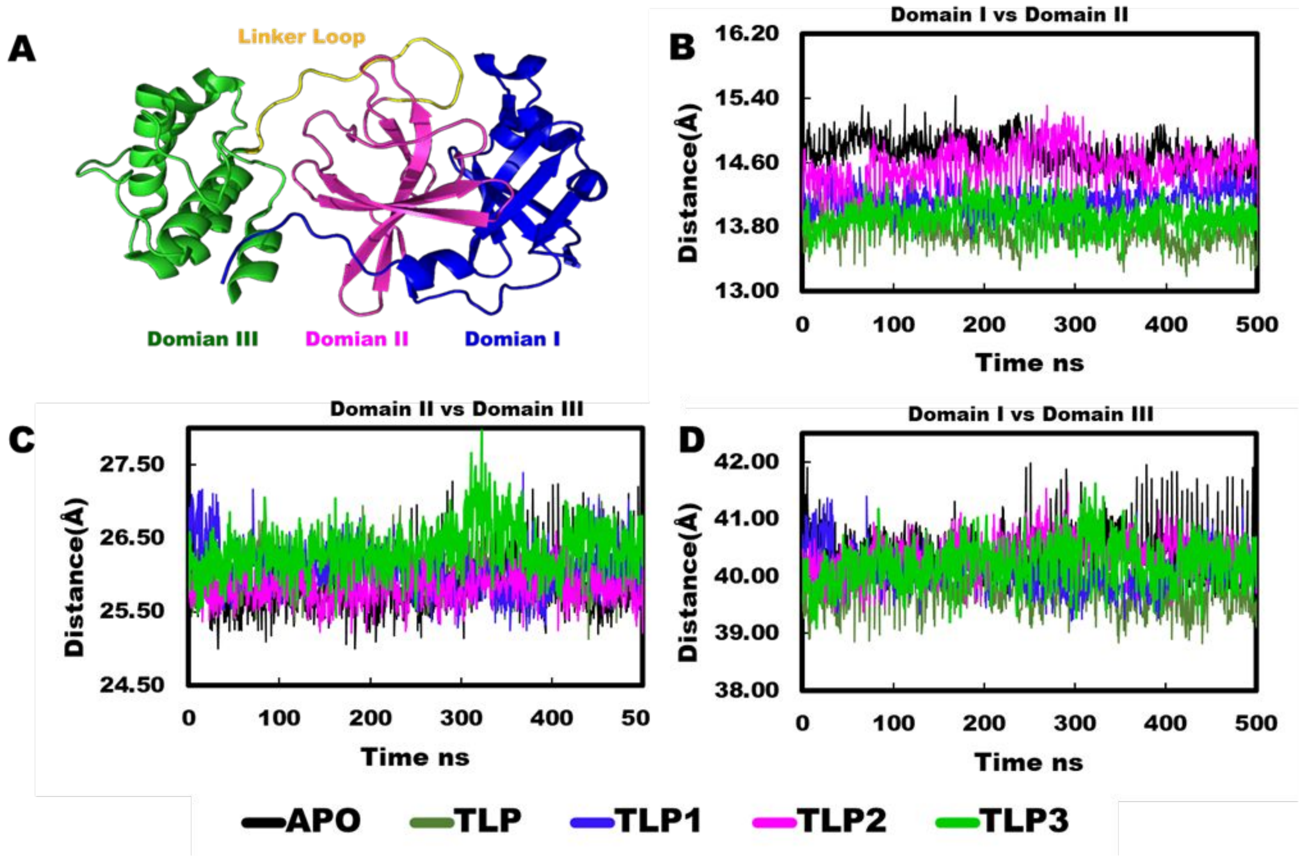

Figure S7: Inter-domain center-of-mass distance analysis from 500 ns molecular dynamics simulations of Mpro in apo form and in complex with different peptides. (A) Structural representation of Mpro showing Domain I (blue), Domain II (magenta), Domain III (green), and the linker loop (Gold). (B) Time evolution of the Domain I–Domain II distance. (C) Time evolution of the Domain II–Domain III distance. (D) Time evolution of the Domain I–Domain III distance. Distances were calculated using Cα atoms from the defined domain residues.

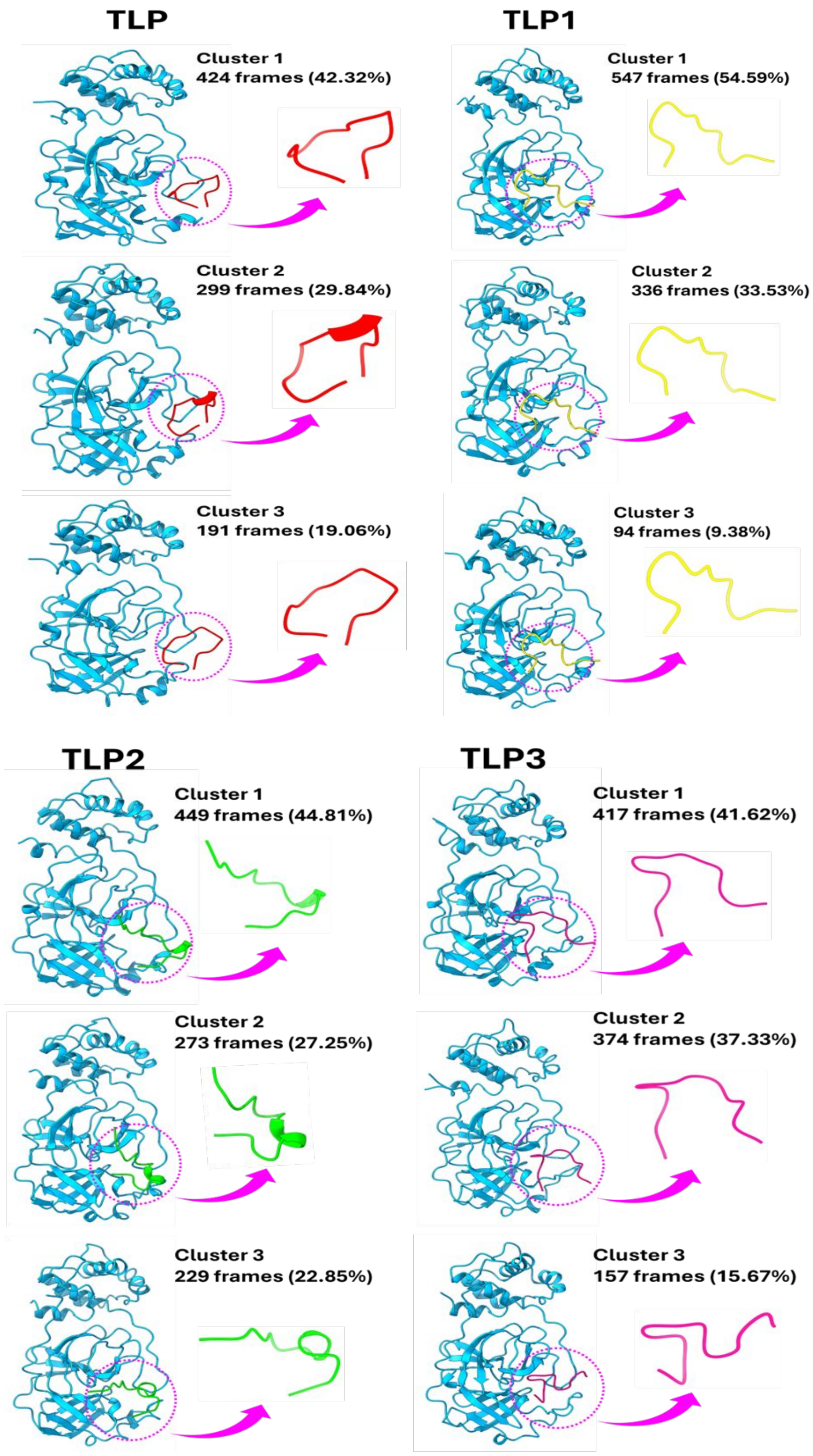
TLP
Cluster 1
424 frames (42.32%)
Cluster 2
299 frames (29.84%)
Cluster 3
191 frames (19.06%)
TLP1
Cluster 1
547 frames (54.59%)
Cluster 2
336 frames (33.53%)
Cluster 3
94 frames (9.38%)
TLP2
Cluster 1
449 frames (44.81%)
Cluster 2
273 frames (27.25%)
Cluster 3
229 frames (22.85%)
TLP3
Cluster 1
417 frames (41.62%)
Cluster 2
374 frames (37.33%)
Cluster 3
157 frames (15.67%)

Figure S8: Representative centroid structures from the three most populated clusters obtained from 500 ns molecular dynamics simulations of each peptide–protein complex. Clusters are ranked by population size, with the number of frames and percentage contribution indicated for each cluster. The protein backbone is shown in cyan, and the bound peptide conformation from each cluster is highlighted in color. Insets show magnified views of peptide conformations from each cluster.
